# The contribution of Sensory Processing Sensitivity and internalized attachment representations on emotion regulation competencies in school-age children

**DOI:** 10.3389/fpsyg.2024.1357808

**Published:** 2024-03-05

**Authors:** Alessandra Sperati, Bianca P. Acevedo, Antonio Dellagiulia, Mirco Fasolo, Maria Spinelli, Giulio D’Urso, Francesca Lionetti

**Affiliations:** ^1^Department of Neurosciences, Imaging, and Clinical Sciences, University of G. d’Annunzio, Chieti-Pescara, Italy; ^2^Department of Psychological and Brain Sciences, University of California, Santa Barbara, Santa Barbara, CA, United States; ^3^Department of Psychology, Salesian University of Rome, Rome, Italy

**Keywords:** Sensory Processing Sensitivity (SPS), attachment, internal working models, internalized attachment relationships, emotion regulation, school-age children, observational study

## Abstract

**Introduction:**

As captured by the individual trait of Sensory Processing Sensitivity (SPS), highly sensitive children perceive, process, and responds more strongly to stimuli. This increased sensitivity may make more demanding the process of regulating and managing emotions. Yet, developmental psychology literature also showed that other variables, as those related to the rearing environment, are likely to contribute to the process of regulating emotions. With the current contribution, we aim to bridge two lines of research, that of attachment studies and that of SPS, by investigating the additive and interactive contribution of SPS and internal working models of attachment representations on emotion regulation competencies in school-aged children.

**Method:**

Participants were *N* = 118 Italian children (mean age: 6.5, SD = 0.58 years, and 51.8% female) with their mothers. Children’s positive attachment representations were rated observationally through the Manchester Child Attachment Story Task procedure during an individual session at school. Mothers reported on children SPS trait and emotion regulation competencies completing the Highly Sensitive Child Scale-parent report and the Emotion Regulation Checklist. We performed and compared a series of main and interaction effect models.

**Results:**

SPS was not directly associated with emotion regulation but it was significantly associated with positive attachment representations in predicting emotion regulation. Highly sensitive children showed poorer emotion regulation when the internalized representations were low in maternal warmth and responsiveness. When driven by sensitive and empathic attachment representation, highly sensitive children showed better emotion regulation than less-sensitive peers, suggesting a for better and for worse effect.

**Discussion:**

Highly sensitive children are not only more vulnerable to adversities but also show better emotion regulation competencies when supported by positive internal working models of attachment relationships. Overall, findings shed light on the link between SPS and attachment and suggest that working for promoting secure attachment relationships in parent–child dyads may promote better emotion regulation competences, particularly in highly sensitive children.

## 1 Introduction

Over the last two decades, empirical evidence has shown that children and adults differ in the degree to which they perceive, process and respond to both positive and negative stimuli, with some showing heightened sensitivity to the environment, for better and for worse (Belsky et al., 2007; Belsky and Pluess, 2009). A reliable psychological marker reflecting such individual differences in responding to stimuli and processing information is the biologically based trait of Sensory Processing Sensitivity (SPS; Aron and Aron, 1997), which is also termed environmental sensitivity (Pluess, 2015). Based on theoretical reasoning and empirical data, individuals with high levels of SPS tend to process information more deeply, and present a stronger emotional reactivity to events (Lionetti et al., 2019; Acevedo et al., 2021; Pluess et al., 2023). According to a neuro-sensitivity hypothesis, the heightened sensitivity, deeper processing and higher reactivity characteristic of SPS seem to stem from a more sensitive and reactive central nervous system (Aron et al., 2012; Pluess et al., 2013; Acevedo et al., 2021). Empirical evidence has also shown that individuals high on SPS tend to be more prone to getting emotionally overwhelmed (for review see Aron et al., 2012; Greven et al., 2019; Lionetti and Pluess, 2023), with potential negative implications for wellbeing and psychological adjustment.

However, some empirical evidence also suggests that an increased sensitivity is not necessarily a risk for emotion regulation issues in children. Instead, it predicts regulation competencies depending on the quality of the environment, such as parenting (Slagt et al., 2018; Lionetti et al., 2019, 2021; Sperati et al., 2022), in a for better or for worse manner (Belsky et al., 2007). Though important from both an applied and theoretical point of view, the evidence is still relatively limited and, most important, the majority of studies explored parenting based on parent-report measures, with the risk of potentially biased results.

The current study sought to fill these gaps by exploring the association between the SPS trait and emotion regulation competencies in children, by examining the moderating role of children’s internalized representations of attachment relationships. The study implemented an observational doll-play procedure—a semi-structured play assessment tool, aiming to evoke, within a standardized setting, patterns of behavior and reaction that originate from an inner working model of attachment relationship (Green et al., 2000). We decided to focus on attachment, as it is considered one of the key variables predicting emotion regulation competencies in children (Waters et al., 2010; Cooke et al., 2019; Tammilehto et al., 2022). Also, we wanted to examine factors that could shed light, and deepen extant knowledge, on variables contributing to emotional competencies in highly sensitive individuals. As such, these results have the potential to inform applications and the practical field about individual and environmental variables that could predispose some children to difficulties in emotion regulation, as well as to flourish in this respect.

### 1.1 Sensory Processing Sensitivity and emotion regulation

Individual differences in the degree to which people perceive, process and respond to both positive and negative environmental stimuli, also defined environmental sensitivity (ES; Pluess, 2015), can be phenotypically captured by the SPS trait (Aron and Aron, 1997). A continuum from low to high sensitivity has been observed within the population, with a quarter of people (∼30%) characterized by a heightened sensitivity, the majority having a medium sensitivity and another sizeable minority characterized by a particularly low sensitivity to environmental stimuli (Lionetti et al., 2019). In line with a Differential Susceptibility perspective (Belsky et al., 2007; Belsky and Pluess, 2009), highly sensitive individuals show a heightened responsiveness not only to negative environment, with a greater risk of mental health problems, but also to nurturing rearing experiences, with a flourishing effect. As further described in the Biological Sensitivity to Context theory (BSC; Boyce and Ellis, 2005), this sensitivity is shaped by early environmental exposures. Brain and gene studies examining adults further suggest that this increased sensitivity has specific structural and functioning correlates in the brain (Acevedo et al., 2014; Pluess et al., 2022), and can be captured by specific genetic variants, such as the dopamine receptor genes (Bakermans-Kranenburg and van Ijzendoorn, 2011), and via a genome wide approach (Keers et al., 2016). Thus, SPS provides a phenotypical marker of such individual differences in responding to the environment, that can be reliably assessed via a range of measures available from the preschool period to adulthood, including self-report, parent-report questionnaires and an observational rating system (Pluess et al., 2018, 2023; Lionetti et al., 2019, 2023; Sperati et al., 2022). SPS refers to a biologically based individual trait that seems to originate from a more reactive nervous system and implies a deeper processing of stimuli, including emotional ones (Acevedo et al., 2014). Several studies have shown associations between heightened SPS and greater activation of brain areas related to both awareness in facial details and emotion recognition (Acevedo et al., 2014; Tabak et al., 2022; Kahkonen et al., in preparation). For example, when exposed to both neutral facial expressions and emotionally-expressive faces, adults high in SPS have been found to present greater activation in areas related to attention and to empathy, respectively (Acevedo et al., 2014). The ability to perceive emotional nuances in the surrounding (i.e., subtleties in facial expression or in prosody), which can be measured with neural activity, is likely the reason for which both adults and school-age children scoring high on SPS have been found to be more empathic and better at emotion recognition (Acevedo et al., 2014; Tabak et al., 2022; Kahkonen et al., in preparation). Moreover, a heightened sensitivity is associated with stronger emotional reactivity, with highly sensitive people (i.e., adolescents and adults) found to experience higher levels of emotions, both positive and negative ones (Lionetti et al., 2019; Weyn et al., 2022; McQuarrie et al., 2023; Pluess et al., 2023). Such stronger emotional reactivity could make it more challenging for highly sensitive individuals to manage emotions, with relevant consequences for socio-emotional adjustment.

Extant studies have shown that SPS is associated with greater risk of mental health problems, including behavioral problems in childhood (Lionetti et al., 2019), and anxiety and depression during adulthood (Liss et al., 2005). At the core of these associations may lie difficulties in coping with heightened emotional arousal. Consistent with this notion, studies involving adults have found high SPS to be associated with poorer emotion regulation strategies, such as limited acceptance of negative affect, higher suppression of feelings and less cognitive reappraisal of emotions (Brindle et al., 2015; Eşkisu et al., 2022). However, empirical evidence on SPS and emotion regulation is still scarce, and according to literature on SPS, for a comprehensive understanding of this link, the role of the rearing environment needs to be considered. Parenting plays a critical role in the development of emotional regulation strategies (Waters et al., 2010; Cooke et al., 2019; Tammilehto et al., 2022), and in line with a differential susceptibility reasoning, it should matter even more for highly sensitive individuals.

### 1.2 Sensory Processing Sensitivity and parenting

Empirical literature focusing on the interaction between SPS and rearing environment in predicting child developmental outcomes has shown that high SPS predicted greater externalizing and internalizing problems in children, as well as difficulties in emotion regulation strategies, especially when exposed to less-than-optimal parenting experiences. According to Slagt et al. (2018), high SPS longitudinally interacted with both changes in negative (i.e., over-reactive, authoritarian, inconsistent parenting) and positive parenting (i.e., responsive and inductive parenting) in predicting changes in externalizing behaviors in preschool children, with findings supporting a differential susceptibility effect. In other words, highly sensitive pre-schoolers showed increased externalizing behaviors when exposed to both decreased positive parenting and increased negative parenting. In contrast, they showed the lowest levels of externalizing behaviors, when high positive parenting was maintained and when negative parenting decreased. Similarly, Lionetti et al. (2019) found observer-rated SPS to moderate the effects of permissive parenting on externalizing behavior in 3-year old children, as well as on internalizing behavioral problems in children at age three and six. Precisely, highly sensitive children showed higher levels of externalizing behavioral problems in a context of permissive parenting but lower levels of externalizing behaviors, as well as internalizing symptoms, similar to their less-sensitive peers, when the permissive parenting was low.

When considering a maladaptive emotion regulation strategy— such as rumination, which is a precursor for depression and internalizing problems (Verstraeten et al., 2011), observer sensitivity ratings in pre-schoolers predicted higher levels of rumination during middle childhood (Lionetti et al., 2021). This, in turn, was related to a higher risk of depression during preadolescence, but only when exposed to a parenting characterized by absence of positive bonding and essential rules, such as the permissive parenting. Similarly, Sperati et al. (2022) found that school-aged children scoring high in SPS were more influenced in their emotion regulation competencies by parenting stress, than their low sensitive peers. In other words, highly sensitive children’s emotion regulation competencies seem not to be necessarily hampered, but to be influenced by the interplay with the quality of the rearing environment and, particularly, parenting. Yet, the extant literature is still scarce, limited to a few studies, and no study explored the environment at an observational level. Self-reports of parenting are widely used and capture, to some extent, parental attitude, and beliefs. However, self-reports can be biased and may not correspond to the way in which a child perceives the parental figure.

### 1.3 Overview of the current study

The current study had two aims. First, to explore the relationship between the individual trait of SPS and emotion regulation competencies. Second, to explore whether this association was moderated by the rearing environment as captured by sensitive and warm internalized representations of maternal caregiving behaviors in the child (i.e., the extent to which the dyadic parent–child relationship was experienced and internalized as responsive, available and empathic). In doing so, we involved a sample of young school-age children, on average 6.5 years of age. According to attachment theory, during the preschool years, children consolidate their internal working models related to their mother–child relationship (Bretherton, 1999). So we considered the first years of school age as an optimal period to investigate internal attachment representations and specifically the extent to which the relationship is represented as warm and empathic. We expected SPS to not be directly associated with emotion regulation. However, when considering the quality of the parenting environment, we expected that high SPS would predict poorer emotion regulation competencies when internalized attachment representations of caregiving behaviors were lower in responsiveness and warmth. At the same time, we expected that high SPS would be associated with stronger emotion regulation abilities, especially among children with more sensitive and warmer internalized attachment representations of their mothers. This hypothesis is in line with attachment theory pointing out that secure representations of parents, such as responsiveness and warmth, promote better emotional regulation (Waters et al., 2010; Cooke et al., 2019; Tammilehto et al., 2022). For low levels of SPS, we expected attachment to play only a trivial, minor role, on children’s emotion regulation competencies.

## 2 Materials and methods

### 2.1 Participants and procedure

Participants were (*N* = 118) school-aged children with their mothers. Children were on average 6.5 years old (age range: 5–8 years old, SD = 0.58) and 51.8% were female. Mothers had a mean age of 37.7 years (age range: 22–55 years old, SD = 6.2) and most were Italian (83%). The majority (92%) lived with the father of the child, and 19% had no other children. The sample was recruited from different schools in Central Italy, from both village and town areas. Recruitment mainly occurred during parents’ evenings at schools during which the research team invited parents to take part in the study and informed consent was obtained from both of the parents who were informed about the study conditions. Children were involved in a quiet, individual play setting with the experimenter during school time. During the session, the experimenter ensured that the child felt comfortable. After obtaining verbal assent from the child, the session started with a 5-min free play moment (e.g., with toys), followed by the administration of the MCAST to assess internalized attachment representations through a doll-play completion method. Each single session lasted a mean of 30 min. The child play sessions were videotaped via a camera provided to the research team, placed in front of the child and the play materials (e.g., house doll), and then videos were coded for attachment representations by two trained researchers, independently. Mothers were invited to fill out paper questionnaire at home.

### 2.2 Measures

#### 2.2.1 Sensory Processing Sensitivity

Children Sensory Processing Sensitivity was assessed with the Highly Sensitive Child–Parent Report scale (HSC-PR; Slagt et al., 2018), recently validated for Italian parents (Sperati et al., 2022). The 12-items aim to capture an increased appreciation for positive environmental stimuli and great attention to subtleties (e.g., “Some music can make my child really happy”; “My child notices when small things have changed in his/her environment”), a lower sensory threshold related to unpleasant sensory arousal (e.g., “loud noises make my child feel uncomfortable”), and a stronger feeling of getting overwhelmed when exposed to potentially adverse experiences (e.g., “my child gets nervous when he/she has to do a lot in little time”). Each item was rated on a 7-point Likert scale ranging from “1 = Not at all” to “7 = Extremely,” with higher scores indicating higher levels of sensitivity. In the current sample, internal consistency of the total score was good (Cronbach’s α 0.77).

#### 2.2.2 Emotion regulation

Children’s emotion regulation competencies were reported by parents with the Emotion Regulation (ER) subscale of the ERC (Shields and Cicchetti, 1997), in its Italian validated version (Molina et al., 2014). The 8-items assess the frequency of behaviors and situationally appropriate affective displays, empathy, and emotional self-awareness with higher scores indicating greater children ability to manage their own emotional arousal. Items are rated by parents on a 4-point Likert scale ranging from 1 = Almost never to 4 = Almost always. In the current sample, internal consistency of the ER subscale approached sufficient values (α = 0.54 dropping out item 23), but consistent with internal reliability shown in the Italian validation of the measure (α = 0.59; Molina et al., 2014).

#### 2.2.3 Internalized positive attachment representations

Children’s representations of caregiving behaviors were observationally assessed with the Manchester Child Attachment Story Task (MCAST; Green et al., 2000; Barone et al., 2009). Based on the attachment theory, MCAST consists of a doll-play completion method and presents children with four story stems that relate to specific attachment stressors (i.e., nightmare, hurt knee, illness, lost in a shopping center) and one preparation vignette (i.e., breakfast). The story stem protagonists are a child and mother figure, implying the dyadic relationship between child and primary caregivers. The MCAST provides both an overall strategy of assuagement (i.e., 4-way attachment classification), as well as an evaluation on single attachment-related dimensional scales related to both child and caregiver behaviors (e.g., warmth, sensitivity, intrusiveness, proximity seeking, self-care behaviors). Because we considered positive emotion regulation competencies as the outcome, we specifically focused on positive caregiving behaviors, such as Responsiveness-Sensitivity and Warmth as perceived by the child. These two scales refer to the caregiver’s physical and emotional responses to the distress of the child, as well as capturing the caregiver’s expression of warm feelings, affect, and empathy.

The Responsiveness-Sensitivity and Warmth dimensions were rated on a 9-point Likert scale ranging from 1 = No evidence of sensitivity to child signals/Cold, uncaring, hostile; to 9 = clear and well timed responsiveness/high levels of warmth, empathy and care. For example, if the child displayed a caregiver who did not respond to the child signaling distress during the doll-playing vignette, (with other goals in the mind), this would be coded as 1 for Responsiveness-Sensitivity. If the child represented the mother as cold and uncaring, with or without overt violence and hostility, this was coded as a 1 or 2. As the two positive dimensions were strongly associated with each other (*r* = 0.83), we computed a mean score. The higher was the score, the more positive the child’s internalized attachment representation of the caregiving behaviors. Inter-rater reliability was tested on 30 encodings. Raw agreement on Responsivity-Sensitivity and the Warmth dimension was 85% (Cohen’s κ =0.72, *p* < 0.001).

### 2.3 Data analysis

#### 2.3.1 Descriptive statistics and linear correlations

We first explored the percentage of missing values and whether the missing data were below 10% to adopt listwise deletion. Linear correlations between all study variables were computed to investigate whether children’s SPS, emotion regulation competencies, internalized attachment representations, age and gender were associated with each other. We considered associations to be low when Pearson’s *r* was around 0.10 or less, medium if *r* varied around 0.30, and large if *r* was higher than 0.50 (Cohen, 1988, 1992). We further calculated the *r* critical for the current sample size.

#### 2.3.2 Main and interaction effect models

Next, we ran and compared a series of main and interaction effects models between SPS and the warm attachment representation variable in predicting emotion regulation across three steps. We first ran a linear regression model considering SPS as a predictor of children emotion regulation (i.e., model 1 = emotion regulation ~ SPS). Second, we ran a main effect model adding to SPS positive internalized caregiving behaviors as a predictor variable (i.e., model 2 = emotion regulation ~ SPS + positive internalized caregiving behaviors). Lastly, we performed the interaction model including positive internalized caregiving behaviors as the moderating variable (i.e., model = 3: emotion regulation ~ SPS X positive internalized caregiving behaviors), to investigate whether SPS predicted emotion regulation depending on levels of warm and sensitive attachment representations.

To evaluate whether the inclusion of the interaction term improved the model’s prediction term, we compared the main effects and interaction effect models using the *R*^2^ (i.e., the total variance of the outcome variable accounted by the model), the AIC (Akaike, 1974) indices, and the Akaike weights (Burnham and Anderson, 2002). According to AIC criterion, the lower the value, the better the model was at predicting data, while for *R*^2^ and Akaike weights, ranging from 0 to 1, the higher the value, the better the model was at describing data accurately (Wagenmakers and Farrell, 2004; Vandekerckhove et al., 2015; McElreath, 2016).

#### 2.3.3 Follow-up exploration

Finally, after selecting the best fitting model, we followed up the interaction effects by adopting a conditional plot. The moderating variable of positive internalized caregiving behaviors was divided in low (below the first 25th quantile) and high levels (above the forth – 75th – quantile). All analyses were run using the statistical software R (R Core Team, 2020). Regression models were run and compared using lm function and AICcmodavg package (Mazerolle, 2017), respectively, and a conditional plot was obtained using ggeffects (Lüdecke, 2018) and ggplot2 (Wickham, 2016) packages.

## 3 Results

### 3.1 Descriptive statistics and linear correlations among variables

As percentage of missing values in the total sample was very low (1.8%), we adopted listwise deletion for handling missing data. Descriptive statistics and linear correlations among all variables are reported in Table 1. Overall, SPS in our sample approached a normal distribution, with the mean value comparable to that found in validation works (mean = 4.8, SD = 0.95) (Slagt et al., 2018; Sperati et al., 2022). Linear correlations showed that children’s SPS was not associated with emotion regulation competencies, with a negative association close to zero (*r* = −0.07), or with positive internalized caregiving behaviors (*r* = −0.08). Trivial associations were also found for SPS and age and gender (*r* = 0.03; *r* = 0.05, respectively). Positive internalized attachment representations were moderately and positively associated with emotion regulation competencies (*r* = 0.28).

**Table 1:** Descriptive statistics and bivariate associations among all study variables (*N* = 111).

	Mean (SD)	1 SPS	2 Positive IWM	3 Emotion Regulation	4 Age
1 SPS	4.8 (0.95)				
2 Positive IWM	4.2 (1.27)	−0.08			
3 Emotion regulation	3.3 (0.33)	−0.07	0.28		
4 Age	6.6 (0.61)	0.03	0.17	0.04	
5 Gender		0.05	0.28	0.15	−0.15

SPS, Sensory Processing Sensitivity; Positive IWM, Positive caregiving behaviors internalized by the child as captured by the MCAST Responsiveness-Sensitivity and Warmth dimensions. Given the sample size, *N* = 111, correlation values greater than 0.18 are significantly different from zero. According to Cohen (1988, 1992): trivial associations: *r* lower than *r* = 0.10; moderate associations: *r* = 25–45; strong association: r equal to or higher than 0.50.

### 3.2 Sensory Processing Sensitivity, warm attachment representations, and their interaction in predicting emotion regulation

Models including only main effects suggested that SPS was not associated with emotion regulation competencies (β = 0.002, *p* = 0.98), while positive internalized caregiving behaviors, in terms of responsiveness to the child’s distress signals and mother’s empathic warm expressions in the relationship, were positively and significantly related to emotion regulation (β = 0.27, *p* = <0.01). When the interaction term was added, a significant effect was found and the model with the interaction effect outperformed the main effect models in predicting better data as suggested by the increase of adjusted *R*^2^ and the Akaike weight, and by the decrease of the AIC criterion (the BIC criteria was comparable between main and interaction effect) (see Table 2 for results of model comparison). Specifically, SPS was significantly associated with positive internalized caregiving behaviors in predicting emotion regulation (β = 1.3, *p* = 0.04). Importantly, regression assumptions were supported as suggested by residuals, approximately normally distributed.

**Table 2:** Comparison of regression models considering SPS and positive internalized parenting behaviors in predicting emotion regulation.

Models	Adjusted *R*^2^	AIC	BIC	delta	Akaike weights
Model 3 (SPS × Positive IWM)	0.09	57	69	0.00	0.72
Model 2 (SPS + Positive IWM)	0.06	59	69	1.91	0.28
Model 1 (SPS)	−0.01	76	84	19.08	0.00

SPS, Sensory Processing Sensitivity; Positive IWM, Positive Inner Working Models – internalized caregiving behaviors.

**Figure 1:**
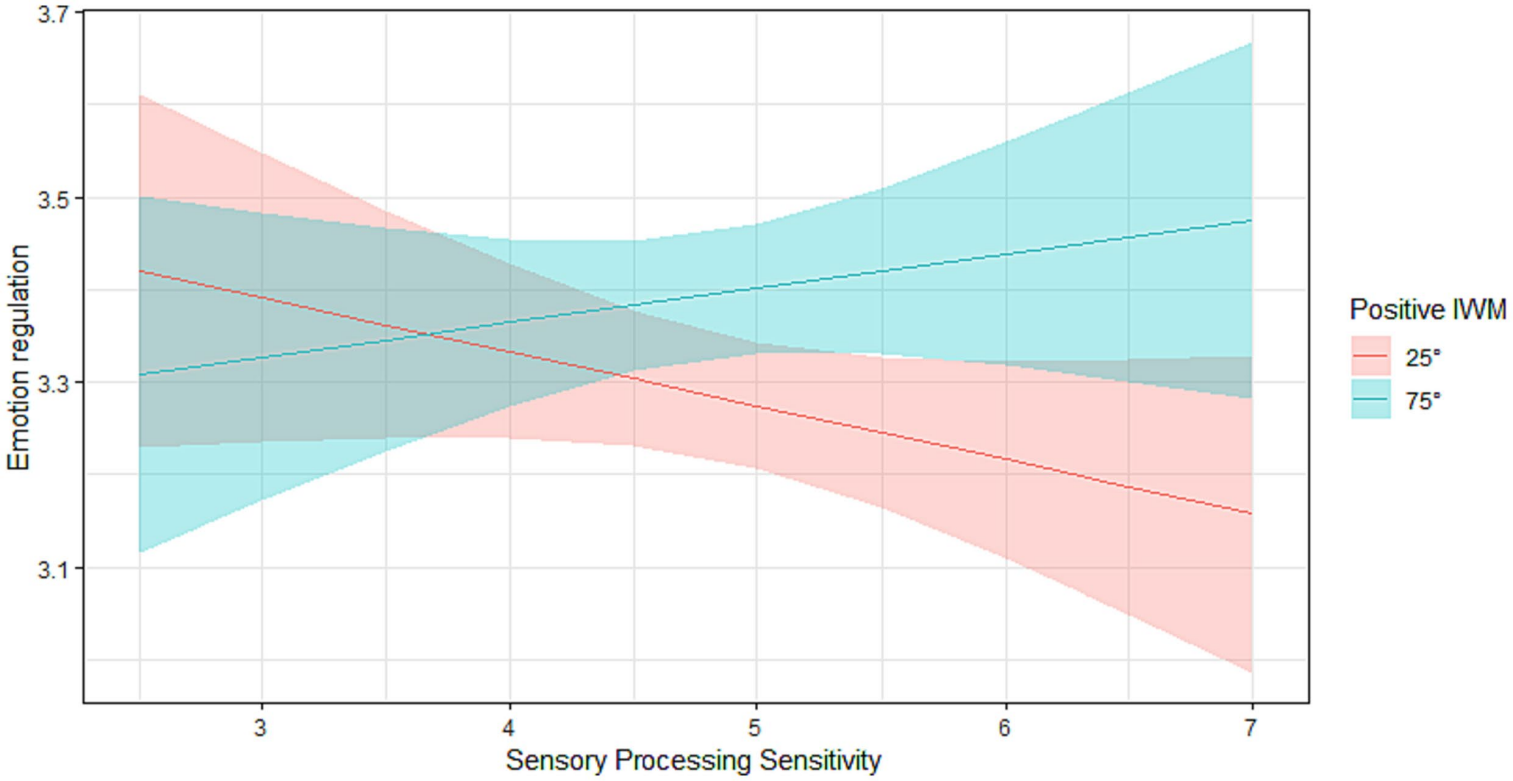
SPS and positive internalized caregiving behaviors, as captured by the MCAST Responsiveness-Sensitivity and Warmth dimensions, in predicting emotion regulation. The moderating variable of positive internalized caregiving behaviors was divided in low (below the first 25th quantile) and high levels (above the forth – 75th – quantile).

To interpret the significant interaction effect, we plotted simple slopes for low (below the first – 25th – quantile) and high (above the forth – 75th – quantile) levels of positive caregiving behaviors as perceived by the child (see Figure 1). The plot suggested that higher SPS was negatively related to children’s emotion regulation competencies when the attachment representation was low in warmth and responsiveness. On the contrary, in a context of positive internalized representations of caregiving behaviors, higher SPS was positively related to the emotion regulation competencies of the child. In other words, children high on SPS showed significantly poorer emotion regulation competencies than less sensitive ones when driven by an internalized attachment representation characterized by low levels of maternal warmth and responsiveness. At the same time, when the internalized caregiving behaviors were high in maternal responsiveness, warmth and empathy; highly sensitive children showed better emotion regulation competencies, compared to less-sensitive children. This suggests that highly sensitive children appear to benefit more from positive internalized relationships with the mother, in comparison with their less-sensitive peers. When levels of SPS were lower, emotion regulation was overall average, irrespective of positive internalized attachment representations.

## 4 Discussion

Some children are characterized by higher levels of Sensory Processing Sensitivity (SPS; Aron and Aron, 1997), and perceive and respond to stimuli, emotional ones included, more intensely. The perception and the experience of strong emotions can pose challenges in regulating emotions, making highly sensitive children more susceptible to feeling emotionally overwhelmed and having behavioral problems, especially when exposed to a suboptimal parenting. For instance, they have been found to show more externalizing behaviors during the preschool period and middle childhood, and internalizing behavior issues during preadolescence — especially when exposed to negative rearing environments such as permissive or inconsistent parenting (Slagt et al., 2018; Lionetti et al., 2019, 2021; Sperati et al., 2022). At the same time, consistent with the differential susceptibility theory, high SPS has been shown to predict increased responsiveness to positive exposures, such as inductive and positive parenting (Slagt et al., 2018). Yet, these studies assessed parenting via self-report measures and most did not explicitly consider the emotion regulation domain that has a fundamental role for psychological wellbeing.

The current study aimed to explore the relationship between SPS and emotion regulation competencies of school-aged children and to investigate the moderating role of observer-rated positive representations of caregiving behaviors, as a marker of the quality of the rearing environment. To this aim, we involved a sample of school-aged children with their mothers recruited in typical neighborhood schools. Children’s positive attachment representations were rated observationally through the MCAST (Green et al., 2000; Barone et al., 2009) during an individual session during school hours, while the mother completed paper questionnaires reporting on their child’s SPS and emotion regulation competencies at home. At a bivariate level, interestingly, SPS was not associated with positive internalized attachment representations. Further investigation is needed in this regard, also considering that among adults SPS has been found to be associated with self-reported insecure attachment styles in romantic relationships (Meredith et al., 2016; Goldberg and Scharf, 2020; Le et al., 2020). As expected, SPS was not directly associated with the children’s emotion regulation competencies. At first glance, this finding seems to contradict previous studies with adults showing that heightened SPS is associated with dysfunctional emotion regulation strategies (e.g., high suppression, low reappraisal). We can speculate that SPS may become a vulnerability factor with age, because of a cumulative risk effect, potentially if the environment has not been positive enough. However, other studies have showed that positive memories of the rearing environment in adults can buffer against this SPS – negative affect association (Lionetti et al., 2024). In other words, for gaining a deeper understanding of how individual differences in SPS influence development, a differential susceptibility perspective should be considered, exploring the interaction between the SPS trait and the environment, in predicting developmental outcomes (Belsky et al., 2007).

Hence, we further explored the relationship between SPS and emotion regulation domain by running an interaction effect model, including the positive internalized caregiving behaviors as the moderating variable. Findings supported a moderating role of the positive internalized attachment representation on the association between SPS and competencies in emotion regulation. The follow-up exploration showed that high SPS was significantly associated with lower emotion regulation competencies when attachment representations were low in warmth, empathic behaviors, and maternal responsiveness to the child’s distress, suggesting that highly sensitive children are more vulnerable than less sensitive ones, to the negative effects of negative attachment representations.

This finding aligns with evidence based on self-reported parenting and suggests that highly sensitive children may find it more difficult to regulate their emotional arousal when they did not experience a warm enough parent–child relationship, as captured by internalized attachment representations. In line with attachment theory, insecure or less-than-optimal internalized attachment representations impact emotion regulation strategies (Waters et al., 2010; Cooke et al., 2019; Tammilehto et al., 2022). This is particularly evident for highly sensitive children who may suffer more when they experience insensitive sensitive, unresponsive and cold representations of maternal caregiving, with low empathy and higher levels of negative effect (i.e., criticism). We can hypothesize that the absence of a clear maternal sensitivity and mirroring of the child’s emotional signals can make it more challenging for children to understand and manage their emotions. This seems to be especially true for highly sensitive children, who tend to feel everything more deeply. This is likely because highly sensitive children, due to their more intense emotional experiences, may require more sensitive and warmer parenting that responds, reflects and contains their strong emotional arousal, and when they do not obtain it, they are less able to regulate their emotional states, compared to less sensitive peers. At the same time, in line with a “for better” effect (Belsky et al., 2007), children scoring high in SPS with positive internalized attachment representations showed better emotion regulation competencies, than their less-sensitive counterparts. This suggests that the experience of maternal behaviors characterized by warmth and lovingness, appropriate and well-timed responsiveness to the child’s emotional signals supports the child in contacting, understanding, and managing his own feelings. Likely due to a greater responsiveness and benefiting from the empathic internalized parenting behaviors, highly sensitive children have been found to be better at managing their emotions and arousal, than less-sensitive peers.

Moreover, our findings are also consistent with results of a study that longitudinally examined the moderating role of another established temperamental trait—effortful control— in association with attachment and brooding rumination strategy in children aged 10 to 14 years (Lindblom and Bosmans, 2022). The study showed that children with high levels of effortful control trait benefited more from low avoidant attachment with the mother (i.e., seeking comfort from their mother), showing lower levels of rumination strategy (Lindblom and Bosmans, 2022). To conclude, results from our study support a differential susceptibility effect, pointing to the “for better and for worse” notion, according to which highly sensitive children are not only negatively affected by suboptimal internalized parenting, but they also benefit disproportionately more from warm, responsive and supportive rearing experiences (Belsky et al., 2007; Belsky and Pluess, 2009).

From a theoretical perspective, these findings contribute to the empirical groundwork aiming at exploring the link between sensitivity and emotion regulation (Lionetti and Pluess, 2023), deepening our understanding of underlying processes that could play a moderating role in SPS and emotion regulation. From a more practical perspective, these findings may inform and provide support for promotional/educational programs for parents about the crucial role of sensitive and warm parenting, particularly for highly sensitive children. Moreover, parenting programs focusing on attachment theory can consider further integrating the role of child’s temperamental and SPS differences into their framework, as highly sensitive children appear to be at greater risk of emotion regulation difficulties, and thus secure dyadic relationships seem to be even more critical for their development, than for children with lower SPS. Additionally, intervention programs targeting children, for example with cognitive behavioral approaches, could benefit from these insights. Practitioners, for example, might consider individual differences in responding to environmental experiences and implement interventions to support highly sensitive children in developing competencies for managing their heightened emotional arousal.

## 5 Strengths and limitations

The literature on SPS suggests that more empirical evidence exploring the underlying processes characterizing the relationship between SPS and emotion regulation is needed. Furthermore, available studies on SPS and self-reported attachment styles are limited to adult samples (Meredith et al., 2016; Goldberg and Scharf, 2020; Le et al., 2020). The current study contributed to fill this gap, providing the first empirical evidence on the moderating role of the observer-rated internalized representation of caregiving behaviors in the association between SPS and emotion regulation among school-age children. The use of a widely used observational method for assessing internalized representations, such as the MCAST, allowed a reliable exploration of the role of internalized positive parenting in influencing emotion regulation competences, especially for highly sensitive children, suggesting relevant insights for both theory and practical field. However, findings should also be considered in light of some limitations. Most importantly, our data on children with high SPS and emotion regulation were based on parent-report questionnaires, and findings could be biased by the mother’s perceptions of her child’s behaviors, lacking objectivity. Future studies should consider multi-method designs, including both observational ratings, to assess children socio-emotional developmental outcomes, and independent informants such as teacher reporting on child behaviors (Pluess and Belsky, 2010). In addition, further assessment with other measures of children’s emotion regulation competencies would be helpful given the sufficient (but not very high) internal reliability of the emotion regulation scale we used in the current work. Moreover, although we were able to rely on a large sample given the observational assessment of the internalized attachment representations, further exploration of this effect would be helpful, considering that our results had overall modest effect sizes.

## 6 Conclusion

The current study provided the first empirical evidence on the moderating role of the observer-rated internalized attachment representations of caregiving behaviors, in the association between SPS and emotion regulation competency in a sample of school-aged children. Findings suggest that SPS was not directly associated with children’s emotion regulation competencies. However, when exploring the moderating role of positive (warm, responsive, and sensitive) caregiving behaviors, internalized by the child, high SPS significantly predicted better emotion regulation when the parental caregiving behaviors were higher in warmth and emotional responsiveness. At the same time, high SPS predicted poorer emotion regulation competency when the attachment representations of caregiving were low in sensitivity (warmth and responsiveness) to the child’s distress. Consistent with previous evidence, parenting characterized by low responsiveness to the child’s needs and caregiving behaviors low in warmth (e.g., mother’s responses to child’s signals of distress are absent or poorly timed, cold or delayed, or lacking empathy and lovingness), and its related internalized representation, might represent a risk factor for difficulties in emotion regulation, especially for children with high sensitivity, which experience stronger emotional reactivity and thus are in greater need of adequate emotion mirroring and containment. At the same time, it seems that having a mother with a parenting style high in warmth and responsiveness is more impactful for highly sensitive children in developing better emotion regulation competencies. This study represents a first contribution to the knowledge on underlying mechanisms characterizing the hypothesized association between SPS and emotion regulation (Lionetti and Pluess, 2023), and provides practical insight for the field and parents, to support highly sensitive children in coping with their more intense arousal.

## Data availability statement

The raw data supporting the conclusions of this article will be made available by the authors, without undue reservation.

## Ethics statement

The studies involving humans were approved by the Department of Psychology, Salesian University of Rome. The studies were conducted in accordance with the local legislation and institutional requirements. Written informed consent for participation in this study was provided by the participants’ legal guardians/next of kin.

## Author contributions

AS: Data curation, Formal analysis, Methodology, Writing – original draft. BA: Writing – review & editing. AD: Supervision, Writing – review & editing. MF: Writing – review & editing. MS: Writing – review & editing. GD’U: Writing – review & editing. FL: Conceptualization, Methodology, Supervision, Writing – review & editing.
